# Elicitor-induced transcription factors for metabolic reprogramming of secondary metabolism in *Medicago truncatula*

**DOI:** 10.1186/1471-2229-8-132

**Published:** 2008-12-22

**Authors:** Marina A Naoumkina, XianZhi He, Richard A Dixon

**Affiliations:** 1 Plant Biology Division, Samuel Roberts Noble Foundation, 2510 Sam Noble Parkway, Ardmore, OK 73401, USA

## Abstract

**Background:**

Exposure of *Medicago truncatula *cell suspension cultures to pathogen or wound signals leads to accumulation of various classes of flavonoid and/or triterpene defense molecules, orchestrated via a complex signalling network in which transcription factors (TFs) are essential components.

**Results:**

In this study, we analyzed TFs responding to yeast elicitor (YE) or methyl jasmonate (MJ). From 502 differentially expressed TFs, *WRKY *and *AP2/EREBP *gene families were over-represented among YE-induced genes whereas *Basic Helix-Loop-Helix *(*bHLH*) family members were more over-represented among the MJ-induced genes. Jasmonate ZIM-domain *(JAZ*) transcriptional regulators were highly induced by MJ treatment. To investigate potential involvement of *WRKY *TFs in signalling, we expressed four *Medicago WRKY *genes in tobacco. Levels of soluble and wall bound phenolic compounds and lignin were increased in all cases. *WRKY W109669 *also induced tobacco *endo-1,3-β-glucanase *(*NtPR2*) and enhanced the systemic defense response to tobacco mosaic virus in transgenic tobacco plants.

**Conclusion:**

These results confirm that *Medicago WRKY *TFs have broad roles in orchestrating metabolic responses to biotic stress, and that they also represent potentially valuable reagents for engineering metabolic changes that impact pathogen resistance.

## Background

Terrestrial plants, having a sessile life style, have evolved a variety of active defense mechanisms to protect themselves against pathogens and pests. For example, in response to pathogen attack the plant can undergo localized hypersensitive cell death associated with synthesis of antimicrobial molecules termed phytoalexins and a range of so-called pathogenesis-related (PR) proteins [1-5]. Induction of plant defense responses occurs through a highly complex signalling network. Transcription factors (TFs) are essential components of these signalling pathways, by controlling the regulation of expression of genes encoding PR proteins and enzymes involved in the synthesis of defense-related compounds [6,7].

We recently described how global profiling of transcripts, associated with metabolic profiling, has revealed details of the mechanisms underlying the induction and accumulation of various classes of flavonoid and triterpene defense molecules in cell cultures of the model legume *Medicago truncatula *[8-13]. In particular, we have shown that the mechanisms by which the cells respond to two different elicitors, the pathogen mimic yeast elicitor (YE) and the wound signal methyl jasmonate (MJ), differ not only in the final end products accumulating, but also in the nature of the underlying signal transduction pathways [13]. The differences are most likely orchestrated by rapid induction of different sets/combinations of transcription factors [5,14-16].

WRKY proteins belong to a large family of transcriptional regulators which contain the conserved amino acid sequence WRKYGQK together with a zinc-finger-like motif [17]. Members of the WRKY TF family are involved in transcriptional regulation associated with plant immune responses [18] and development [19]. In the past decades, significant progress has been made on the characterization of WRKY proteins involved in regulation of plant defense responses [20]. Over-expression of *WRKY *genes in transgenic plants has shown that some are able to increase the production of PR proteins and to modulate resistance to phytopathogens [21,22]. Most of these studies have utilized the model crucifer *Arabidopsis thaliana*. Few studies have addressed the transcriptional control of defense-related secondary metabolism in legumes.

We here describe the families of *Medicago *TFs that are induced by YE or MJ in cell suspension cultures of the model legume *M. truncatula*, with particular focus on members of the *WRKY *family. Our results show that different classes of transcriptional regulators are activated by YE and MJ in *Medicago *cell cultures, and that heterologous expression of selected *Medicago *WRKY proteins in transgenic tobacco enhances typical defense responses such as PR protein induction and accumulation of soluble and wall bound phenolic compounds.

## Results and discussion

### Classes of TFs regulated by YE or MJ in *M. truncatula *cell suspension cultures

**Figure 1:**
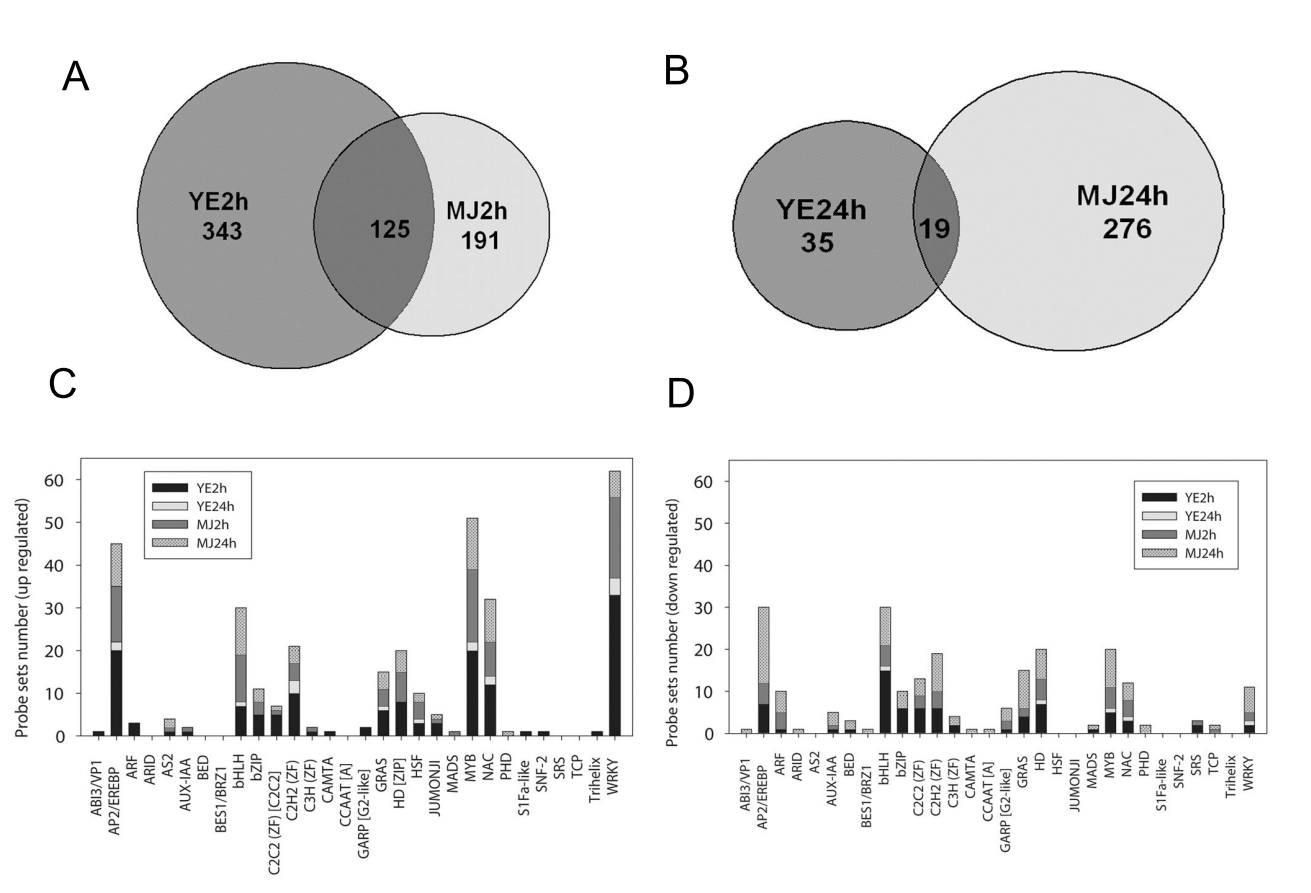
**TFs regulated by YE or MJ in *M. truncatula *cell suspension cultures**. Venn diagrams showing the numbers of transcription factors induced by YE or MJ at 2 h (*A*) and 24 h (*B*) of treatment. Up- (*C*) and down-regulated (*D*) transcription factors are classified according to [23].

More than 1,350 TFs have been identified in the *M. truncatula *genome to date [23], and new classes of plant TFs are still being discovered [23,24]. To provide a global analysis of TFs that are transcriptionally regulated by YE or MJ, we performed transcript profiling using Affymetrix *Medicago *arrays to compare 2 h and 24 h elicited samples to corresponding controls. The array contains over 61,200 probe sets: 32,167 *M. truncatula *EST/mRNA-based and chloroplast gene-based probe sets; 18,733 *M. truncatula *IMGAG and phase 2/3 BAC prediction-based probe sets; 1,896 *M. sativa *EST/mRNA-based probe sets; and 8,305 *Sinorhizobium meliloti *gene prediction-based probe sets. Genes encoding 502 TFs were differentially expressed in *M. truncatula *cell suspension cultures treated with YE or MJ (Additional file 1). However, the pattern of activation of TFs by YE or MJ was different (Figure 1A, B). After 2 h of treatment, 343 TFs were up-regulated by YE, 191 by MJ, and 125 by both (Figure 1A). At 24 h, the differences between the two treatments were greater; the number of TFs up-regulated by YE dropped, but MJ up-regulated TFs increased and only 19 were up-regulated by both treatments (Figure 1B).

TFs are classified based on their DNA-binding motifs. To investigate which TF classes were regulated by YE or MJ treatments, we used the recently published legume TF classification [23]. Figure 1C, D shows the up- and down-regulated TFs distributed in annotated classes, not including putative TFs. Five gene families were over-represented among the up-regulated TFs, namely the *AP2/EREBP*, *bHLH*, *MYB*, *NAC *and *WRKY *families (Figure 1C). *AP2/EREBP *and *NAC *families are specific to plants [25]. *WRKY *TF genes were rapidly and highly up-regulated by YE. Among the 65 YE-induced *WRKY *probe sets, more than half were up-regulated at 2 h post-elicitation, with fewer being more highly expressed than in the controls at 24 h. MJ induced only 20 and 7 *WRKY *probe sets at 2 h and 24 h, respectively. Notably, the *WRKY *most strongly induced by YE (TC109669; almost 600-fold change) was induced five times higher than the most strongly MJ-induced *WRKY *(TC108267; 115-fold change) (Table 1). A group of 20 probe sets corresponding to *AP2/EREBP *gene family members was up-regulated at 2 h, followed by a strong reduction in their expression at 24 h of YE treatment. Thirteen and ten *AP2/EREBP *probe sets were induced by MJ at 2 h and 24 h, respectively. The *WRKY *TFs were over-represented among the twelve TFs that were most highly up-regulated (more than 20-fold) by YE (Table 1).

**Table 1:** The most highly expressed transcription factors in *Medicago *cell cultures exposed to YE or MJ

Probesets	YE2h	YE24h	MJ2h	MJ24h	Accession	Family
Highest induced in response to YE						
Mtr.11349.1.S1_at	**594.20**	0.92	**3.67**	0.87	TC109669	*WRKY*
Mtr.42577.1.S1_s_at	**168.56**	1.43	**2.66**	**0.43**	TC111875	*WRKY*
Mtr.12149.1.S1_at	**65.32**	**0.39**	**9.81**	0.52	TC112312	*WRKY*
Mtr.43241.1.S1_at	**41.54**	1.59	1.31	**0.04**	TC94874	*WRKY*
Mtr.15568.1.S1_s_at	**20.99**	1.81	1.06	0.65	747.m00011	*WRKY*
Mtr.15018.1.S1_at	**136.00**	0.73	**219.23**	**2.50**	773.m00019	*MYB_HD-like*
Mtr.16873.1.S1_s_at	**124.00**	1.04	**0.21**	**0.46**	887.m00014	*MYB*
Mtr.38413.1.S1_at	**23.15**	**2.84**	**2.01**	**4.03**	TC102745	*MYB*
Mtr.16212.1.S1_at	**83.05**	1.68	**0.47**	0.73	861.m00015	*AP2/EREBP*
Mtr.5395.1.S1_at	**36.35**	1.96	**3.27**	1.42	BE320193	*AP2/EREBP*
Mtr.12511.1.S1_at	**35.39**	**4.11**	**3.52**	1.94	TC95045	*HSF*
Mtr.15278.1.S1_s_at	**24.97**	**3.10**	1.44	**0.28**	780.m00021	*C2H2 (ZF)*
Highest induced in response to MJ						
Mtr.22988.1.S1_at	**0.29**	0.67	**239.60**	**9.66**	1643.m00042	*bHLH*
Mtr.27133.1.S1_at	1.69	**0.48**	**137.66**	**18.73**	AW561111	*bHLH*
Mtr.51379.1.S1_at	1.63	1.50	**65.23**	**53.94**	751.m00006	*bHLH*
Mtr.43316.1.S1_at	0.51	0.93	**60.15**	**68.28**	TC95049	*bHLH*
Mtr.12392.1.S1_at	1.49	1.40	**22.41**	**13.65**	TC94630	*bHLH*
Mtr.18769.1.S1_at	**0.48**	1.01	**33.97**	**46.25**	1047.m00031	*HD_ZIP*
Mtr.15018.1.S1_at	**136.00**	0.73	**219.23**	**2.50**	773.m00019	*MYB_HD-like*
Mtr.10896.1.S1_s_at	**2.56**	1.44	**115.88**	**51.18**	TC108267	*WRKY*
Mtr.40890.1.S1_at	**4.95**	1.97	**83.14**	**83.64**	TC108268	*WRKY*
Mtr.20232.1.S1_at	**3.22**	0.53	**24.38**	**12.81**	1207.m00022	*AP2/EREBP*

Accessions include IMGAG Annotated Medicago BACs [95] and DFCI Medicago Gene Index Release 8.0 (January 19, 2005) [98]. Numbers represent fold change – elicited/control; only significant data are highlighted in bold.

The *Basic Helix-Loop-Helix (bHLH) *family of eukaryotic TFs [26] was more pronouncedly over-represented among the MJ-induced genes. Seven and one *bHLH *probe sets were induced at 2 h and 24 h after YE treatment, respectively, and 11 probe sets were up-regulated by MJ at 2 h and 24 h post-treatment. The *bHLH *gene family was over-represented among the ten highest MJ-induced TF genes (Table 1).

Four gene families, *AP2/EREBP*, *bHLH*, *HD *and *MYB*, were over-represented among the down-regulated TFs (Figure 1D). The *AP2/EREBP *gene family was more over-represented among the genes down-regulated by MJ, with seven *AP2/EREBP *probe sets down-regulated by YE at 2 h. Five and 18 probe sets were down-regulated by MJ at 2 h and 24 h, respectively. In contrast, the *bHLH *gene family was more over-represented among the TFs that were down-regulated by YE. Fifteen and one *bHLH *probe sets were down-regulated by YE, and five and nine were down regulated by MJ, at 2 h and 24 h respectively.

The mechanism of transcriptional regulation by MJ was largely unknown until the recent discovery of a novel family of transcriptional regulators called jasmonate ZIM-domain (*JAZ*) proteins [27-29]. These function as repressors of MJ-regulated transcription in *Arabidopsis*.*JAZ *proteins are normally bound to TFs and inhibit their activity. It has been shown that *MYC2 *specifically recognizes the G-box sequence in the promoter of the *JAI3 *(*JAZ*) gene [27]. In response to wounding, jasmonoyl-isoleucine [30] stabilizes the interaction between the SCF^COI1 ^(Skip/Cullin/F box) E3 ubiquitin ligase complex [31,32] and *JAZ*. The *JAZ *proteins are probably modified by ubiquitination and destroyed by the 26S proteasome [27,28]. Degradation of *JAZ *repressors liberates the TFs that induce jasmonate-responsive transcriptional changes. Twelve proteins have been identified in *Arabidopsis *[27,28]. Homology within this family is confined to two domains, a 'TIFY' motif that contains the conserved amino acid pattern TIF(F/Y)XG [33], and a carboxy-terminal domain [27]. Outside these domains the sequence similarity is weak, and the proteins do not contain any known DNA-binding domain [27]. So far, *JAZ *proteins have been identified only in plant species [33]. *Arabidopsis JAZ *genes were quickly and specifically induced by jasmonate treatment or constitutively expressed in untreated plants overexpressing *MYC2 *under control of the 35S promoter [27]. These findings suggest that a negative feedback mechanism may limit the response after initial jasmonate perception [27].

We identified seven genes in *M. truncatula*, represented by 12 probe sets, corresponding to *JAZ *proteins that are strongly induced by MJ (Additional file 2). The probe set Mtr.20116.1.S1_s_at corresponding to gene *1101.m00011 *(IMGAG# *AC146572_11*) showed a massive 3,187 fold induction at 2 h after MJ treatment. Transcript analysis of *JAZ 1101.m00010 (AC146572_10) *in different naïve *M. truncatula *tissues through interrogation of the *Medicago *Gene Expression Atlas [34] showed very low expression (Additional file 3A), suggesting that this gene is specifically regulated by MJ. *JAZ *proteins share domain similarity with *ZIM *TFs [27]. Five of the seven *JAZ *genes identified in *M. truncatula *were previously classified as *ZIM *family TFs [23] and two genes were not annotated. The similar response of *JAZ *genes to MJ in *Arabidopsis *and *M. truncatula *may reflect a conserved mechanism of jasmonate regulation between species.

The best characterized TF in jasmonate signaling is *AtMYC2*, which positively regulates genes involved in the wound response but negatively regulates genes involved in pathogen defense [14]. *ERF1 *also differentially regulates these two responses, but with the opposite effect to that of *MYC2 *[14,15]. *AtMYC2 *encodes a nuclear localized helix-loop-helix-leucine zipper *bHLH*-type transcription factor [14]. As outlined above, *M. truncatula *TFs of the *bHLH *family were highly induced by MJ and down-regulated by YE (Additional file 1, Figure 1C, D). The gene *1643.m00042 *(*AC141862_14*) was induced 240-fold at 2 h of MJ treatment (Additional file 1). This gene is likely induced specifically by MJ, as its expression was extremely low in naïve *M. truncatula *tissues and only observed in roots following nodulation (Additional file 3B). *AC141862_14 *showed 33% identity and 56% similarity at the amino acid level to *AtMYC2 *(NP_174541). The protein has a nuclear localization signal (ERRRRE), and the gene may be the *M. truncatula *ortholog of *AtMYC2*.

An opposite response to that of *bHLH *TFs was observed for the *Medicago AP2/EREBP *(ethylene responsive) gene family TFs (Additional file 1, Figure 1C, D). The interplay between *bHLH *and *AP2/EREBP *TF families may explain, at the molecular level, how plants select the correct response to pathogen attack or wounding [14].

### Selection of *Medicago *WRKY transcription factors for expression in transgenic tobacco

Among the five families of significantly over-represented TFs induced by YE or MJ, the *WRKY *family predominated (Figure 1). *M. truncatula *cells accumulate isoflavonoid phytoalexins in response to YE [9], and a rapid and massive induction of *WRKY *TF genes is correlated with the induction of genes involved in the central phenylpropanoid pathway and the downstream steps in the biosynthesis of the isoflavonoid phytoalexin medicarpin [9,13]. Roles of TFs in plant defense have been demonstrated in several species including *Arabidopsis*, tobacco, parsley and other plants [35-43]. However, no gain- or loss-of-function studies to characterize *WRKY *proteins from *Medicago *species have been reported to date.

In order to investigate the potential involvement of Medicago *WRKY *TFs in the regulation of the phenylpropanoid pathway, we decided to over-express candidate genes in tobacco. Because of the large amount of redundancy among transcription factor families, a gain-of-function approach was chosen as it might be more likely to yield a detectable phenotype [25,44].

**Figure 2:**
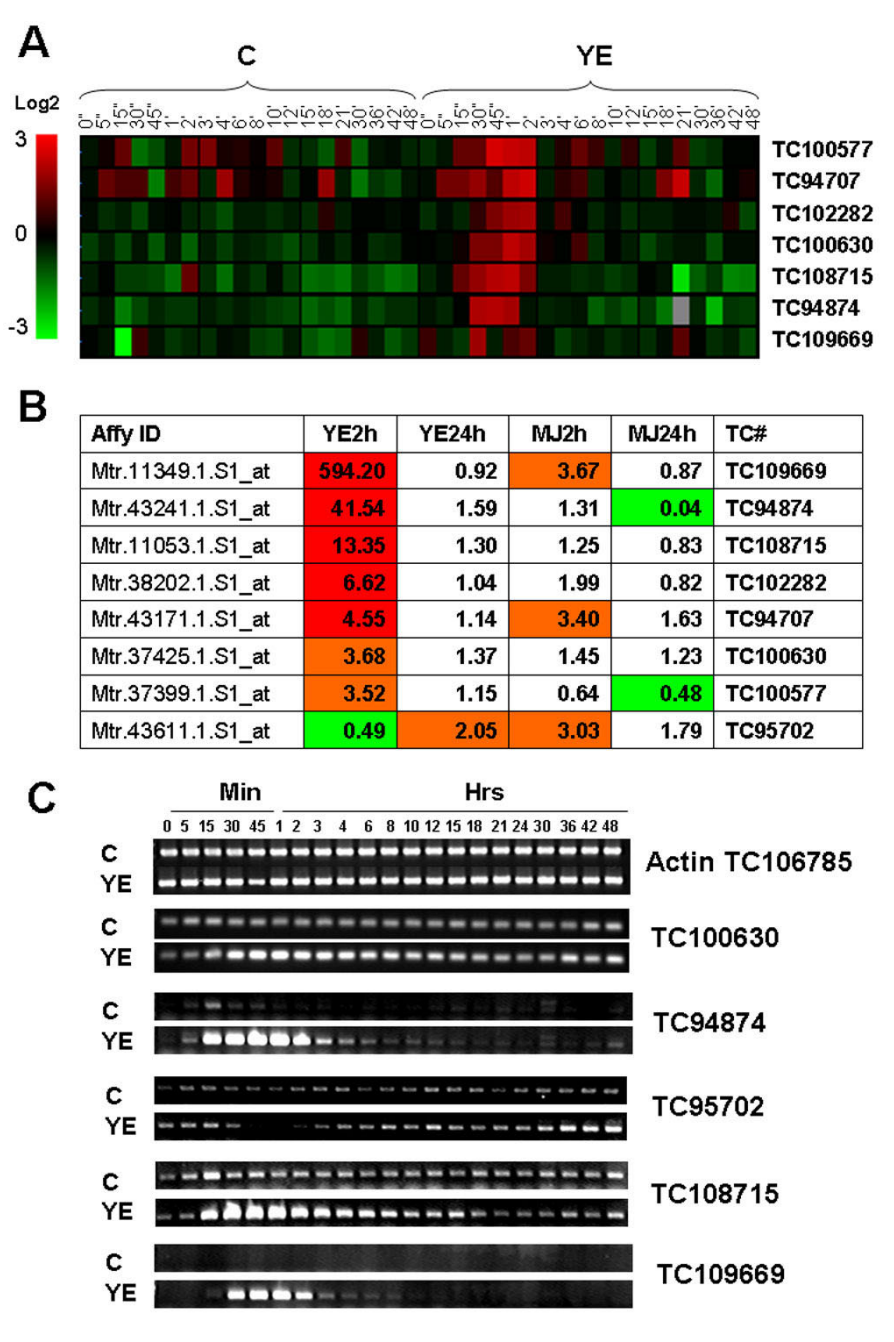
**Expression of WRKY transcription factors in *M. truncatula *cell cultures**. A, induction of *WRKYs *by YE as revealed by oligonucleotide array analysis. The double apostrophes represent minutes and the single apostrophes represent hours. B, *WRKY *transcript levels in YE and MJ treated cells determined by Affymetrix array analysis. C, Detailed time course for *WRKY *gene transcript levels in response to YE, as determined by RT-PCR. Actin is shown as loading control.

Selection of *WRKY *TFs was based on the pattern of their transcriptional induction by YE. The heat map in Figure 2A shows detailed induction kinetics of seven *WRKY *genes induced by YE as revealed by oligonucleotide array analysis. This approach gives lower reproducibility than the Affymetrix arrays, but allows for analysis of more time points due to its much lower cost. Many of the *WRKY *genes were rapidly induced by as early as 15 min after treatment, and their transcript levels were reduced after 2 h post-elicitation. Using the more sensitive Affymetrix microarray technique, the transcript levels of the *WRKY *genes were quantified at 2 h and 24 h after YE or MJ treatment (Figure 2B). The most strongly expressed *WRKY*, corresponding to tentative consensus (TC) 109669, was up-regulated 594-fold in response to YE. The expression kinetics of several of the *WRKY *genes, including the one down-regulated by YE, were confirmed by non-quantitative RT-PCR (Figure 2C) and the results further validated by semi-quantitative RT-PCR (Additional file 4).

On the basis of the above expression patterns, four *Medicago WRKY *TCs (100577, 100630, 108715 and 109669) were selected for cloning and transformation into tobacco. It should be noted that three of these (100577, 100630 and 108715) were not the most strongly induced according to quantitative Affymetrix data (Table 1); these *WRKYs *were selected based on oligonucleotide microarray data before the Affymetrix platform for *Medicago *was available. Genomic sequences were available for *W100577 *(AC174357_2) and *W109669 *(CT963079_8). The second most strongly induced *WRKY*, TC111875, might be a splice variant of CT963079_8. The amino acid sequence of *W100630 *showed 50% identity to Arabidopsis *WRKY6 *(NP_564792.1).

Full length cDNA sequences were available for three of the above genes. The cDNA clone of TC108715 was truncated at the 5' end, and was completed by RACE PCR. Detailed descriptions of cloning and Gene Bank accession numbers are given in Methods. Amplified cDNA products were cloned into the binary vector pBI121 by replacing the *GUS *gene.

*WRKY *gene family members have low sequence similarity outside the WRKY domain, a 60 amino acid region that is highly conserved. WRKY proteins are classified into three distinct groups based on the number of WRKY domains and differences in their zinc-finger-like motifs [17], and a functional distinction between the domains has been demonstrated [45,46]. A comparative phylogenetic analysis of *Arabidopsis WRKYs *[17] with the selected *Medicago *genes showed that *W108715 *belongs to group I, *W100630 *to group IIb, *W100577 *to group IId, and *W109669 *to group III (Additional file 5). Nuclear localization was predicted for all the selected WRKY proteins with 95% confidence using the 'LOCtree' bioinformatics tool [47]. Additional file 6 shows transcript analysis of the four *WRKY *genes in different plant tissues. *W100630 *(*WRKY *TC100630) is expressed in *Rhizobium*-inoculated roots and at late stages of seed development (Additional file 6A). *W100577 *is expressed relatively highly in all tissues tested, especially in *Rhizobium*-inoculated roots (Additional file 6B). *W108715 *is expressed in petioles, vegetative buds, stems and *Rhizobium*-inoculated roots (Additional file 6C). Very low, almost background transcript levels were detected for *W109669 *(Additional file 6D), suggesting that this gene is most likely involved primarily in defense responses, whereas *W100630, W100577 *and *W108715 *are also expressed during plant development.

### *Medicago WRKY *genes induce phenolic compounds and lignin in transgenic tobacco

**Figure 3:**
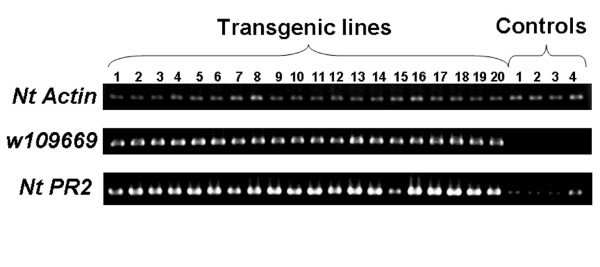
**RT-PCR analysis of *Medicago W109669 *and tobacco *PR2 *transcript levels in transgenic tobacco lines overexpressing *W109669***. Control plants harbored pBI121. Control and transgenic plants of the T_0 _generation were used for analysis. *Actin *is shown as loading control.

**Figure 4:**
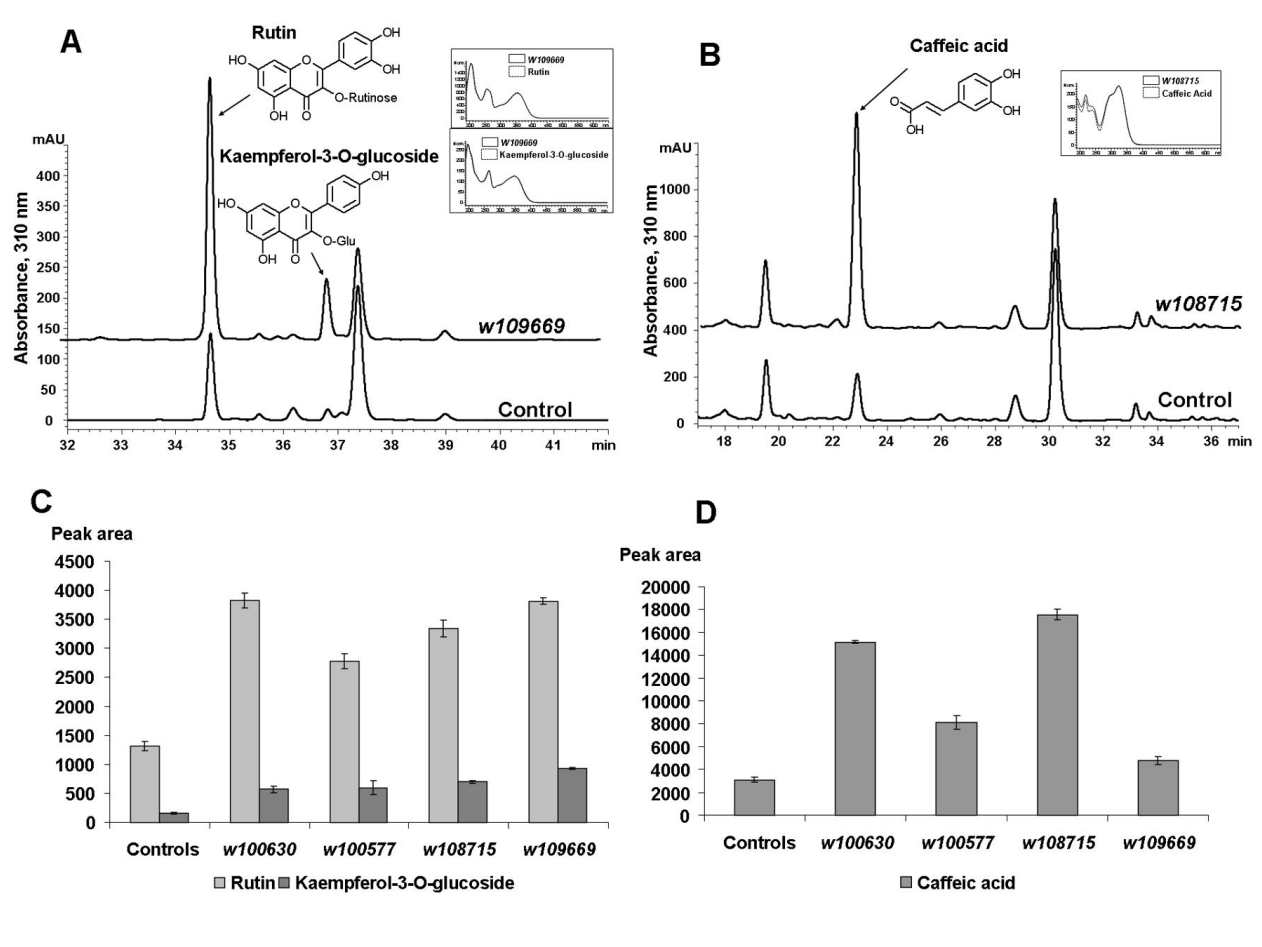
**Levels of phenolic compounds in transgenic tobacco lines expressing *Medicago WRKYs***. Selected regions of HPLC chromatograms show soluble (A) and wall bound (B) phenolic compounds. Insets show the UV spectra of identified peaks. (C) and (D) show relative levels (peak areas) of soluble and wall bound phenolic compounds in control and transgenic lines. Control plants harbor pBI121. Error bars indicate standard error from three biological replicates (control and transgenic lines – T_0 _generation).

Kanamycin resistant plantlets harboring *WRKY *expression constructs regenerated from tissue culture were screened initially by genomic PCR of leaf tissues (data not shown). Transgenic plants did not show significant visible phenotypic changes compared with controls. Most of the transgenic lines showed expression of the transformed *WRKY *gene in the leaf tissue, whereas control lines harboring pBI121 did not (typical data are shown for *W109669 *in Figure 3). Induction of *WRKY *TFs was correlated with accumulation of phenolic compounds in elicited *M. truncatula *cells [9,13]. This suggested examining phenolic compound profiles in transgenic tobacco plants expressing *Medicago WRKYs*. Soluble and wall bound phenolic compounds were therefore extracted from control and *WRKY*-expressing transgenic tobacco lines, and the extracts analyzed by HPLC. Rutin and kaempferol-3-*O*-glucoside were present at higher levels in the soluble fraction from plants expressing any of the four *WRKY *genes than in controls (Figure 4A, C), by more than 2-3-fold for rutin and 3-6-fold for kaempferol-3-*O*-glucoside.

A number of TFs involved in the regulation of flavonoid production have been isolated and reported previously. Generally, these belong to either *MYB *or the basic helix-loop-helix, *MYC*-type families [48,49]. In most studies, co-action of members belonging to both of these gene families was required for the production of anthocyanins in the plant [49-54]. Expression of the maize *LC *(*MYC*-type) and *C1 *(*MYB*-type) genes in the fruit of transgenic tomatoes resulted in a strong accumulation of kaempferol glucoside, but not in the accumulation of anthocyanins, a finding that was explained by insufficient expression of the gene encoding flavanone-3',5'-hydroxylase [55].

Induction of *WRKY *TFs by YE in *M. truncatula *cells correlates with accumulation of the isoflavonoid medicarpin [9,13]. Tobacco does not possess a fully functional isoflavonoid pathway, and ectopic expression of *Medicago WRKYs *in transgenic tobacco shifts the metabolic flux into the accumulation of biosynthetically related flavonol glucosides instead (Additional file 7). The roles of flavonoids in stress- and pathogen-protection are still under investigation, but flavonols may be among the most important flavonoids in this regard [56]. A kaempferol triglucoside was isolated from carnation stems and roots, and was suggested to be an active phytoalexin against the fungal pathogen *Fusarium oxysporum f. sp. dianthi *[57].

**Figure 5:**
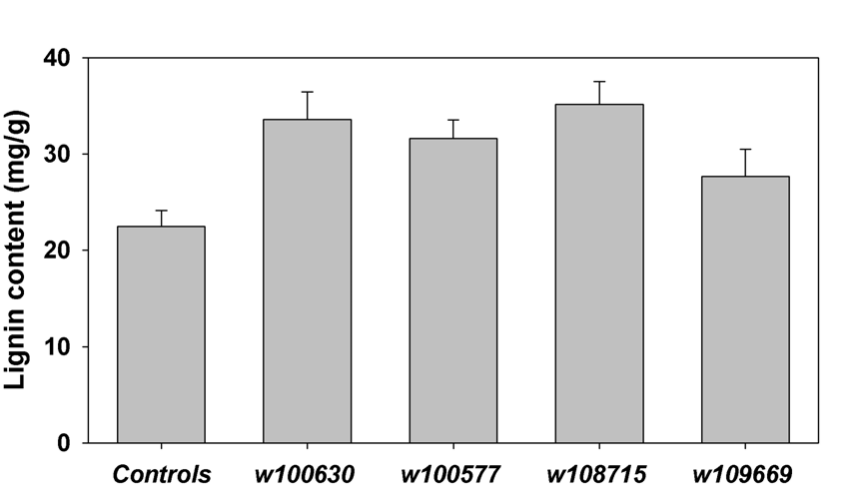
**Lignin content in transgenic tobacco plants expressing *Medicago WRKYs***. Lignin content, determined by the acetyl bromide method, in control and transgenic tobacco plants expressing *Medicago WRKY *genes. Control plants harbor pBI121. Error bars indicate standard error from three biological replicates (control and transgenic lines – T_0 _generation).

The level of wall bound caffeic acid increased in transgenic tobacco lines expressing three of the four *WRKY *genes (Figure 4B, D), by more than 4-fold in lines expressing *W100630 *and *W108715*. Increased levels of caffeic acid were correlated with increased lignin content, as determined by the acetyl bromide method, in these lines (Figure 5). Elevated lignin accumulation was previously reported in transgenic rice lines over-expressing *OsWRKY89 *[40], and has also been shown in *M. truncatula *suspension cells in response to YE, but not MJ (Lei, Z at al. unpublished results). Induced lignification is one of several plant defense responses to pathogen attack and wounding [58-61]. Transgenic rice over-expressing *OsWRKY89 *also showed enhanced ultraviolet tolerance and disease resistance, suggesting that *OsWRKY89 *plays an important role in responses to biotic and abiotic stress. Our similar results from transgenic tobacco plants expressing *Medicago WRKYs *suggest that these TFs have broad roles in orchestrating metabolic responses that impact stress tolerance.

### Induction of lignin biosynthetic genes in *M. truncatula*

Affymetrix microarray analysis showed that several genes encoding enzymes involved in lignin biosynthesis were induced in *M. truncatula *cells exposed to YE or MJ (Additional file 8). Most of the probe sets representing lignin pathway genes were up-regulated at 2 h of YE treatment (Additional file 8). Two cytochrome P450 enzymes, cinnamate 4-hydroxylase (C4H) [62,63] and coumarate 3-hydroxylase (C3H) [62,63], as well as hydroxycinnamoyl CoA: shikimate hydroxycinnamoyl transferase (HCT) [64], caffeic acid 3-*O*-methyltransferase (COMT) and cinnamyl alcohol dehydrogenase (CAD), were induced by YE, but not by MJ. Caffeoyl-CoA 3-*O*-methyltransferase (CCoAOMT) and three probe sets representing cinnamoyl Coenzyme A reductase (CCR) were up-regulated by both YE and MJ, but induction by YE was higher. All ferulate 5-hydroxylase (F5H) probe sets showed high expression at later time points after MJ treatment, and two showed delayed induction by YE. One of the probe sets, iMsa.1740, showed 98% nucleotide identity to the functionally characterized *F5H-K10 *of alfalfa [62,63]. F5H catalyzes hydroxylation at the C_5 _position of coniferaldehyde and coniferyl alcohol in S monolignol biosynthesis [65], and down-regulation of *F5H *significantly reduced the yield of syringyl monomers in transgenic alfalfa lines [62]. However, lignin accumulation was not observed in MJ treated *M. truncatula *cells [66].

Laccases and peroxidases are involved in dehydrogenative polymerization of monolignols into lignin [65]. Five probe sets of genes encoding laccases were induced by YE at 2 h of treatment, and one set was also induced by MJ (Additional file 8). From 49 probe sets of putative peroxidases, 32 were up-regulated by YE and 16 by MJ. The correlation of WRKY TFs with activation of lignin biosynthetic genes and increased levels of lignin in *M. truncatula *cells, coupled with their ability to induce lignin in transgenic tobacco, suggests involvement of *WRKY *TFs in induced lignification responses.

### Expression of *W109669 *induces *PR2 *and enhances the response to TMV in transgenic tobacco plants

The importance of phenylpropanoid compounds in plant disease responses has been much discussed [67-70]. Induction of phenylalanine ammonia-lyase (PAL) and downstream enzymes of the phenylpropanoid pathway is associated with viral-induced necrosis in tobacco [71], and suppression of *PAL *compromised systemic resistance in tobacco plants infected with tobacco mosaic virus (TMV) [67]. Tobacco may have four *PAL *genes, which appear to be expressed in most tissue types [72-75]. Expression of *PAL3 *[75] was not altered from control levels in tobacco lines expressing *Medicago WRKY *genes (data not shown). Other *PAL *genes might therefore be involved in the biosynthesis of phenolic compounds in tobacco expressing *Medicago WRKY *genes.

Salicylic acid (SA) is a signal molecule in the establishment of both local and systemic acquired resistance (SAR) in tobacco [76]. Plants expressing a bacterial salicylate hydroxylase gene demonstrated no accumulation of *pathogenesis-related *(*PR*) gene 1 transcripts [77], suggesting that *PR1 *expression is dependent on production of SA. Rather than directly measuring SA levels in tobacco expressing *Medicago WRKY *genes, we determined the levels of *NtNPR1 *[78] and *NtPR2 *[79] transcripts. Differences in *NtNPR1 *transcript levels were not detected in any plants expressing any of the *WRKY *genes. However, *NtPR2 *was constitutively expressed in transgenic plants expressing *W109669 *(Figure 3). *NtPR2 *is an acidic, extracellular, endo-1,3-β-glucanase) [79]. A defensive role for β-1,3-glucanases in plants has been suggested by the observation that β-1,3-glucanases and chitinases are coordinately induced in response to pathogens [79]. β-1,3-Glucans are important structural components of fungal cell walls, and *in vitro *evidence shows that β-1,3-glucanase in combination with chitinase has a direct fungicidal action on some phytopathogenic fungi [80]. β-1,3-Glucanases may also act indirectly by releasing elicitors from fungal cell walls that can subsequently stimulate phytoalexin accumulation in the host plant [81].

Because *W109669 *induces *NtPR2 *transcripts in tobacco, we examined systemic disease resistance of transgenic plants expressing either *W109669 *or *W108715 *(which does not induce *NtPR2*) by inoculation with tobacco mosaic virus (TMV) U1 strain. Plants were pre-inoculated with virus on the lower leaves, and secondary inoculations were performed on upper leaves five days later. As shown in Additional file 9, the sizes of the secondary lesions formed in *W109669 *expressing lines were much smaller than in controls or in lines expressing the other *WRKY *genes, indicating that *W109669 *enhanced SAR in tobacco.

The observation that ectopic expression of any one of four *WRKY *TFs promoted increased levels of flavonols in tobacco, that three out of the four also induced caffeic acid levels, and that some, but not all, induce SAR raises questions as to whether these results reflect functional redundancy among family members, or simply non-specific effects due to high level ectopic expression in a heterologous species. Functional redundancy is common not only for TFs [44,82]. For example, when 86% of the 19,427 predicted *Caenorhabditis elegans *genes were knocked down, only 10% of the resulting mutants exhibited any phenotype [83]. For more then 40 *Arabidopsis WRKY *knock-down mutants, phenotypes were rarely observed [82]. However, it is clear that WRKY TFs are crucial regulators of defense responses in *Arabidopsis*. For example *AtWRKY53 *was identified as a positive regulator, and *AtWRKY58 *as negative regulator, of SAR [84]. *AtWRKY70 *has been shown to play an important role in determining the balance between SA-dependent and JA-dependent defense pathways [21,85]. Ectopic expression of TFs at high level may have pleiotropic effects [86]. For example, expression of the Arabidopsis MYB transcription factor, TT2, in *Medicago *hairy roots resulted in up-regulation of over 400 probesets [87]. Only 45 of them overlapped with probesets preferentially expressed in *M. truncatula *seed coat, the organ in which TT2 is naturally expressed, indicating that a large number of genes were non-specifically up-regulated by TT2. However, massive accumulation of proanthocyanidins was observed in the hairy roots expressing TT2 [87], confirmed the potential of using TFs for metabolic engineering.

Although ectopic over-expression of four *Medicago WRKYs *in tobacco led to similar chemical phenotypes, only *W109669 *induced *PR2 *expression and enhanced SAR. Thus, ectopic over-expression in a heterologous system can reveal differences between *WRKYs *in relation to the expression of defense-associated marker genes and the response to certain pathogens. These are useful observations from a biotechnology perspective, but do not of themselves indicate precise functions for these genes in *M. truncatula*. Analysis of recently available *M. truncatula *Tnt1 retrotransposon insertion lines [88] may provide a loss-of-function approach to address this issue. Irrespective of considerations of *in vivo *function, the fact that ectopic expression of *Medicago WRKY genes *increases the levels of phenolic compounds and lignin in tobacco highlights the value of such genes for engineering improved chemical defenses or increasing levels of health-beneficial antioxidant polyphenols in agricultural crops.

## Conclusion

Challenging *M. truncatula *cell suspension cultures with YE or MJ leads to accumulation of various classes of flavonoid or triterpene defense molecules. Complex signal transduction network controls such processes, of which TFs are essential components as master regulatory proteins controlling the transcriptional cascade. We have observed opposite regulation of *AP2/EREBP *and *bHLH *TF families in response to YE or MJ that may explain, at the molecular level, how plants select the correct response to pathogen attack or wounding. Strong induction of WRKY TFs by YE suggests that they may have a role in signaling in response to this pathogen mimic. Expression of four *Medicago WRKY *TF genes in transgenic tobacco increased phenolic compounds and lignin, suggesting that these TFs have broad roles in orchestrating metabolic responses implicated in biotic stress tolerance. Expression of *W109669 *in transgenic tobacco enhanced viral tolerance, suggesting a potential role of this TF in triggering an additional genetic cascade for disease resistance independent of lignin and phenolic production.

## Methods

### Plant Material

Details of the initiation and elicitation of *M. truncatula *Gaerth 'Jemalong' (line A17) cell suspension cultures have been provided elsewhere [9,11,13]. Transgenic plants of *N. tabacum *cv Xanthi NN were grown in 4.5 inch diameter pots containing "Professional blend" soil (Sun Gro Horticulture, Bellevue, WA) at a temperature of 20°C/19°C (day/night). Plants were fertilized at time of watering using a commercial fertilizer mix (Peters Professional 20-10-20 (N-P-K) General Purpose, The Scotts Company, Marysville, OH).

### Gene constructs and plant transformation

Sequences of full length cDNA clones representing *WRKY *genes can be accessed in GenBank, accession numbers: *W100577 *– EU526033, *W100630 *– EU526034, *W108715 *– EU526035, and *W109669 *– EU526036. Full length cDNA clones were available for *W100577*, *W100630*, and *W10966*. The cDNA clone of *W108715 *was truncated at its 5' end; this part of the sequence was recovered by RACE-PCR using the BD SMART RACE cDNA amplification kit (BD Biosciences Clontech Inc., Palo Alto, CA) according to the manufacturer's protocol. *WRKY *genes were cloned, by PCR using primers with a BamHI site at the 5'-end and a SacI site at the 3'-end (Additional file 10), into the BamHI/SacI sites of the binary vector pBI121 (GB Accession AF485783) [89] to replace the *GUS *gene.

The binary vector constructs including pBI121 as negative control were introduced into *Agrobacterium tumefaciens *strain C58C1 by electroporation. Agrobacteria harboring the plasmid were confirmed by colony PCR and used for transformation of *N. tabacum *cv Xanthi NN. Leaf disc transformation of tobacco was performed as previously described [90].

### RNA isolation and non-quantitative and semi-quantitative RT-PCR

Total RNA was isolated from 0.5 g of frozen, ground tissue of *M. truncatula *suspension cells or *N. tabacum *leaves using 5 ml of Tri-Reagent (Molecular Research Center, Cincinnati, OH) following the manufacturer's protocol. Three μg of total RNA was used in a first strand synthesis using Ready-To-Go RT-PCR Beads (Amersham Biosciences Corp, Pittsburgh, PA) in a 50 μl reaction with oligo-dT primers according to the manufacturer's protocol. Two μl of the first strand reaction was then PCR amplified for 30 cycles at 68°C annealing temperature using Takara Ex Taq (Fisher Scientific Company, Palatine, IL) according to the manufacturer's protocol. The PCR products were analyzed on an agarose gel.

Semi-quantitative RT-PCR was performed using a Quantum RNA 18S internal standard kit (Ambion Inc., Austin, TX) according to the manufacturer's protocol. Each RT-PCR reaction was repeated with three independent biological replicates. PCR products were separated in a 1% agarose gel and stained with Syber Green (Invitrogen Inc., Carlsbad, CA). The fluorescence signal was captured using a UVP Bioimaging system (UVP, Inc., Upland, CA). Analysis of signal intensity of products was performed with Image Quant TL software (Amersham Biosciences, Pittsburgh, PA). Data were normalized according to 18S internal standard.

The sequences of oligonucleotide primers used in RT-PCR experiments are given in Additional file 11.

### Microarray analysis

DNA microarray analysis was performed utilizing oligonucleotide microarrays representing 16,086 TC sequences and Affymetrix Medicago genome arrays with 61,000 probe sets as described previously [9,13]. For the oligonucleotide arrays, a reference design was employed in which all RNA samples for both control and elicited cells were compared to RNA from a separate batch of non-elicited cells. Three biological replicates were used. The Amino Allyl cDNA Labelling Kit (Ambion Inc, Austin, TX) was used to label 25 μg of total RNA following the manufacturer's protocol. Cy3 dye (Amersham Biosciences Corp, Piscataway, NJ) was used for labelling the reference RNA and Cy5 for the experimental samples. The arrays were read with a ScanArray 4000 scanner (Packard, Palo Alto, CA) at 10 μm resolution and variable photomultiplier tube voltage settings to obtain maximal signal intensities. The fluorescence intensity for each flour and each element on the array was captured using GenePix Pro 4.1 (Axon, Union City, CA). Normalization of Cy3 and Cy5 signal was performed by adjusting the signal intensities of the two images using the Lowess (sub-grid) method of the GeneTraffic software, and the local background was subtracted from the values of each spot on the array. Statistical analysis (ANOVA) of normalized data was performed using GeneSpring software as described previously [9].

For experiments using the Affymetrix GeneChip^® ^*Medicago *Genome Array (Affymetrix, Santa Clara, CA), RNA samples were prepared from cells exposed to YE or MJ for 2 h or 24 h, along with the corresponding unelicited controls. Two biological replicates, with analytical duplicates, were used for minimal statistical treatment, and mean values for each treatment were divided by the corresponding control baseline values. Full details of the experimental procedures and statistical analysis have been presented elsewhere [13].

The complete Affymetrix dataset is publicly available at ArrayExpresss [91], and the oligo array data are available via the DOME database at the Virginia Bioinformatics Institute [92].

### Analysis of phenylpropanoid compounds

Tobacco leaves (1 g fresh weight) were ground in liquid N_2_. Extraction of soluble and wall bound phenolic compounds were as described previously [93,94]. Separation and quantification of phenolic compounds were as described previously [94].

### Determination of lignin content

Lignin content of transgenic tobacco leaves was determined by the acetyl bromide method using ~30 mg extractive-free material [95,96]. A molar extinction coefficient of 17.2 [95] was used for samples from all the control and transgenic lines.

### Tobacco mosaic virus inoculation

Purified TMV U1 strain was mechanically inoculated onto leaves of tobacco plants at the 10 leaf stage using carborundum powder (Sigma, St Louis, MO). The lower leaves were inoculated with 100 μl per leaf of TMV solution (1.27 ng/μl). Five days later, when the local lesions appeared on the lower leaves, the upper leaves were inoculated with the TMV solution and the resulting lesions measured 4–5 days after the secondary inoculation.

## Abbreviations

AP2/EREBP: AP2/ethylene responsive element binding protein; bHLH: Basic Helix-Loop-Helix; MJ: Methyl jasmonate; PR: Pathogenesis-related; TC: Tentative consensus (from MTGI v 8.0); TMV: Tobacco mosaic virus; TF: Transcription factor; YE: Yeast elicitor; ZF: Zinc finger.

## Competing interests

The authors declare that they have no competing interests.

## Authors' contributions

MN, XH, and RAD designed research; MN and XH performed research;

MN analyzed data; MN and RAD wrote the paper.

## Supplementary Material

Additional file 1 ***M. truncatula *TFs differentially expressed in response to YE or MJ**. This table shows microarray analysis of transcription factor genes whose transcripts are either up-regulated or down-regulated in *M. truncatula *cell cultures exposed to either yeast elicitor or methyl jasmonate. TFs were classified according to [23]. Accessions include IMGAG Annotated Medicago BACs [97] and DFCI Medicago Gene Index Release 8.0 (January 19, 2005) [98]. Numbers represent fold change – elicited/control, only significant data are color coded (p-value < 0.05); green, fold change less than or equal to 0.5; orange – more than or equal to 2.0 and less then 4.0; red – more than or equal to 4.0. Click here for file

Additional file 2 **Affymetrix analysis of *M. truncatula *genes putatively encoding JAZ proteins**. This table shows microarray analysis of predicted *M. truncatula *JAZ genes which were strongly induced by methyl jasmonate. *M. truncatula *gene predictions were based on sequence similarity to Arabidopsis JAZ genes [27,28]. Click here for file

Additional file 3 **Affymetrix microarray expression analysis of *Medicago genes JAZ AC146572_11 *and *bHLH AC141862_14***. This figure shows expression levels of two transcriptional regulators, *JAZ AC146572_11 *and *bHLH AC141862_14*, in different naïve *M. truncatula *tissues. The *Medicago *genes were: (A) *AC146572_11 *(homolog to *AtJAZ1*); (B) *AC141862_14 *(homolog to *AtMYC2*). Transcript levels were measured in the different tissues shown, including seeds at various stages of development (numbers refer to days post pollination, dpp) and nodules (Nod) derived from *Rhizobium*-inoculated roots at various times (numbers refer to days post-inoculation, dpi). Root-0d – roots at 0 dpi (control for nodule developmental series). Nodule – nodules from 4 weeks old plant. VegBud – vegetative buds (apical and lateral meristem regions). Error bars indicate standard deviation from three biological replicates. Data were mined from the Medicago Gene Atlas [34]. Click here for file

Additional file 4 **Semi-quantitative RT-PCR analysis of *WRKY *transcript levels**. The data show representative changes of *WRKY *transcripts in response to yeast elicitation based on semi-quantitative RT-PCR analysis. Data represent the fold change in transcript level in response to YE as compared to unelicited control. Error bars indicate standard deviation from three biological replicates. Click here for file

Additional file 5 **Phylogenetic analysis of *Arabidopsis *and *M. truncatula WRKY *proteins based on their DNA-binding *WRKY *domain**. This figure shows a phylogenetic tree of *Arabidopsis *and *M. truncatula WRKY *proteins, based on their DNA-binding *WRKY *domains. The amino acid sequences of the *Medicago WRKY *sequences reported here were compared with those of published *Arabidopsis WRKY *TFs [17] and additional sequences available online [99]. Amino acid sequences from the single WRKY domain of group II and III members or the C-terminal WRKY domain of group I members were aligned using the MegAlign program in the DNASTAR Lasergene package software (DNASTAR, Inc., Madison, WI). The ClustalW method with BLOSUM series of protein weight matrix was used for alignment. The numbers above the branches are bootstrap values from 1000 replicates. Click here for file

Additional file 6 **Affymetrix microarray analysis of the tissue specificity of expression of *WRKY *TFs**. This figure shows *WRKY *gene expression profiles in different naïve *M. truncatula *tissues. Genes were: (A) *W100630*; (B) *W100577*; (C)*W108715*; (D)*W109669*. Transcript levels were measured in the different tissues shown, including seeds at various stages of development (numbers refer to days post pollination, dpp) and nodules (Nod) derived from *Rhizobium*-inoculated roots at various times (numbers refer to days post-inoculation, dpi). Root-0d – roots at 0 dpi (control for nodule developmental series). Nodule – nodules from 4 weeks old plant. VegBud – vegetative buds (apical and lateral meristem regions). Error bars indicate standard deviation for three biological replicates. Data were mined from the Medicago Gene Atlas [34]. Click here for file

Additional file 7 **Scheme of the flavonol biosynthesis pathway**. This figure shows a scheme of the flavonol biosynthesis pathway in *Medicago*. Enzymes are: CHS, chalcone synthase; CHR, chalcone reductase; F3H, flavanone-3-hydroxylase; IFS, isoflavone synthase; 2HID, 2-hydroxyisoflavanone dehydratase; FLS, flavonol synthase; GT, glucosyltransferase. Click here for file

Additional file 8 **Affymetrix analysis of *M. truncatula *genes involved in the lignin pathway that are induced in response to YE or MJ**. This table shows Affymetrix microarray analysis of genes involved in the lignin pathway which were either up-regulated or down-regulated in *M. truncatula *cell cultures exposed to either yeast elicitor or methyl jasmonate. Click here for file

Additional file 9 **Enhanced TMV resistance in transgenic tobacco lines overexpressing *W109669***. The data shown an analysis of the sizes of the secondary lesions formed in transgenic tobacco lines overexpressing *W109669 *after inoculation with tobacco mosaic virus. Bars show the size (diameter) of secondary lesions on TMV infected control and transgenic tobacco lines expressing *Medicago W108715 *or *W109669*. Control plants harbored pBI121. Error bars indicate standard errors for the size of lesions from three control and transgenic lines of the T_1 _generation. Click here for file

Additional file 10 **Primers for cloning *Medicago WRKY *TFs**. This table presents the sequences of the gene-specific primers used for cloning *Medicago WRKYs*. Click here for file

Additional file 11 **Primers for gene-specific RT-PCR analysis of transcripts in *M. truncatula *cell cultures and transgenic *N. tabacum *lines**. This table presents the sequences of gene-specific primers complementary either to *Medicago *or tobacco genes used for RT-PCR analysis. Click here for file
