# Supplementary figures and images for: Elicitor-induced transcription factors for metabolic reprogramming of secondary metabolism in Medicago truncatula
Source: BMC Plant Biol. 2008 Dec 22;8:132. doi: 10.1186/1471-2229-8-132 (PMC2628384; doi:10.1186/1471-2229-8-132)

**A*****JAZ 1101.m00010 / AC146572\_10 (Mtr.49627.1.S1\_at)***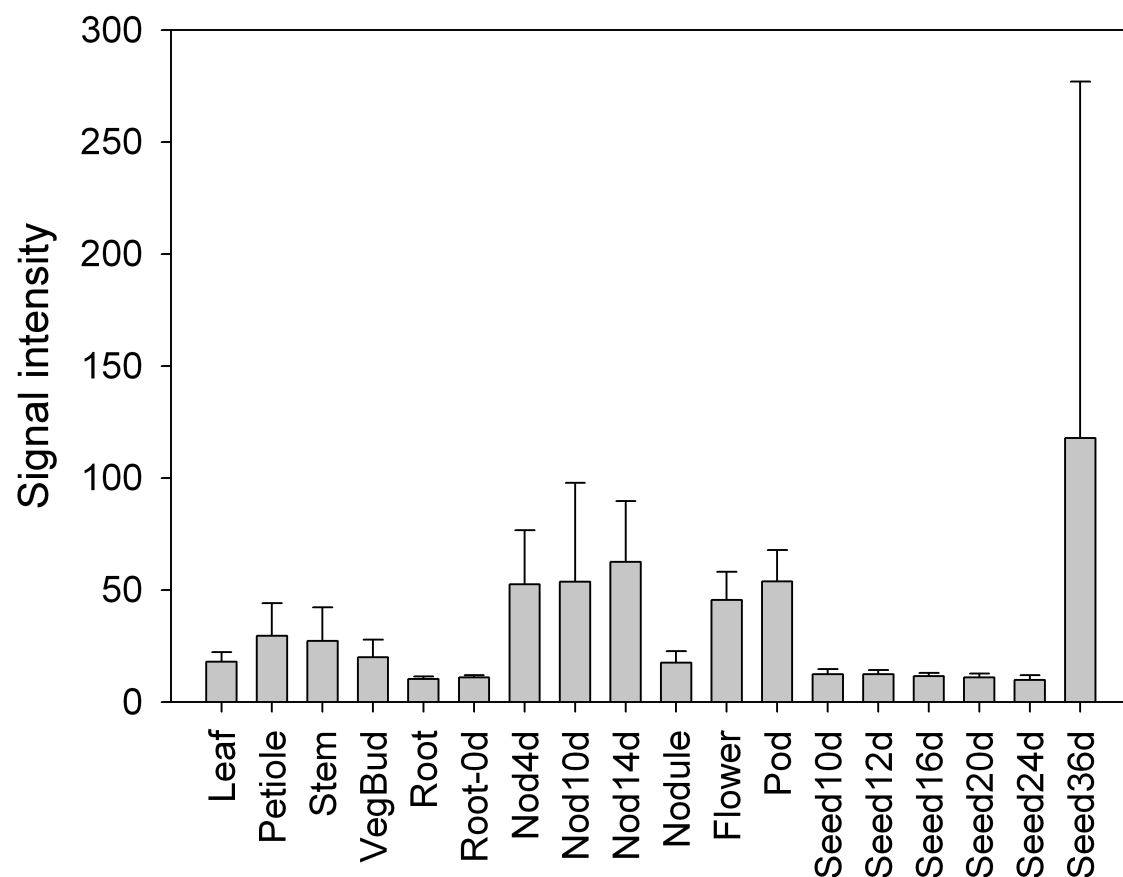**B*****bHLH 1643.m00042 / AC141862\_14 (Mtr.22988.1.S1\_at)***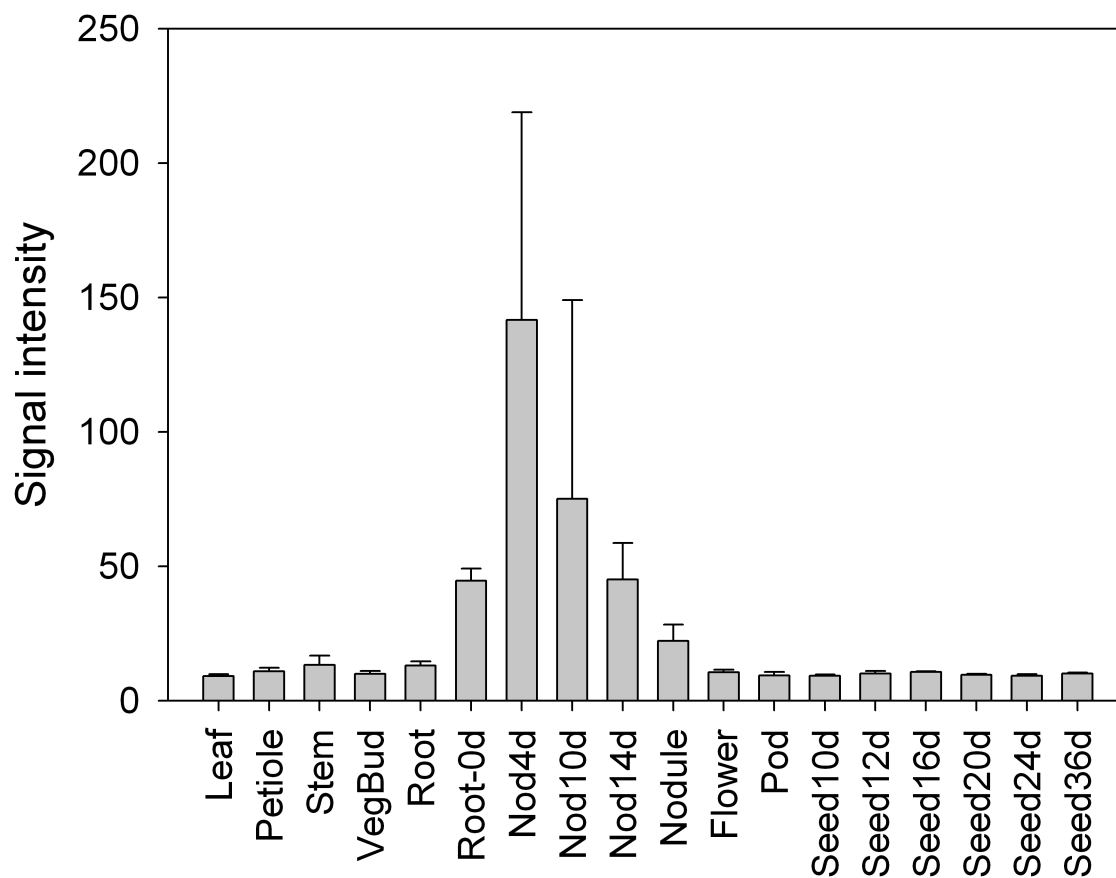

Supplement: Additional file 3 — Affymetrix microarray expression analysis of Medicago genes JAZ AC146572_11 and bHLH AC141862_14. This figure shows expression levels of two transcriptional regulators, JAZ AC146572_11 and bHLH AC141862_14, in different naïve M. truncatula tissues. The Medicago genes were: (A) AC146572_11 (homolog to AtJAZ1); (B) AC141862_14 (homolog to AtMYC2). Transcript levels were measured in the different tissues shown, including seeds at various stages of development (numbers refer to days post pollination, dpp) and nodules (Nod) derived from Rhizobium-inoculated roots at various times (numbers refer to days post-inoculation, dpi). Root-0d – roots at 0 dpi (control for nodule developmental series). Nodule – nodules from 4 weeks old plant. VegBud – vegetative buds (apical and lateral meristem regions). Error bars indicate standard deviation from three biological replicates. Data were mined from the Medicago Gene Atlas [34]. [file 1471-2229-8-132-S3.pdf]

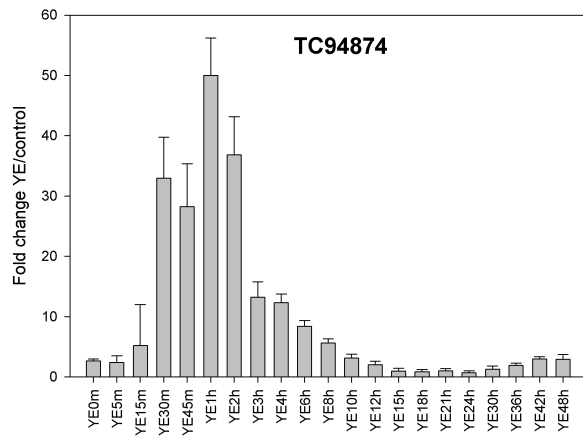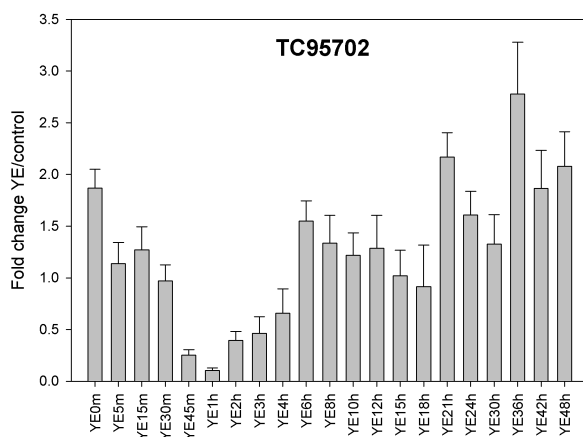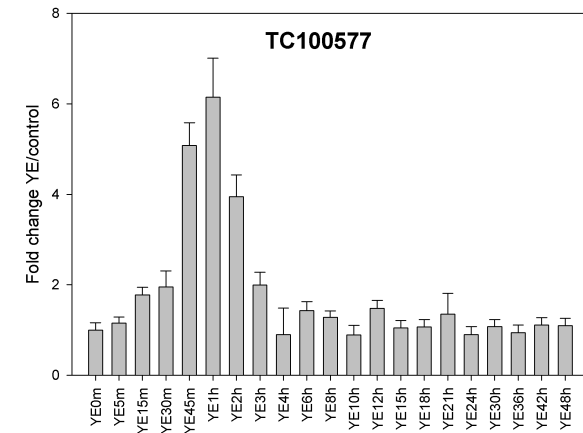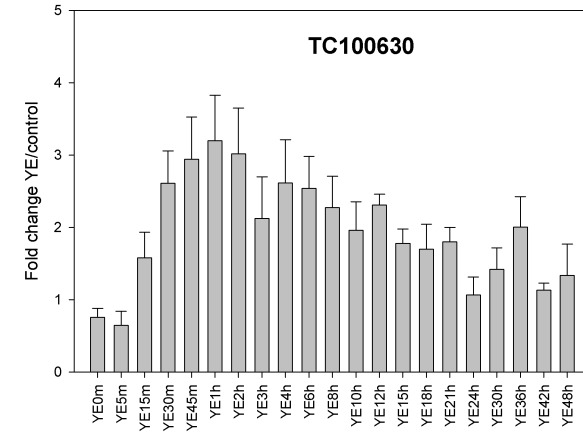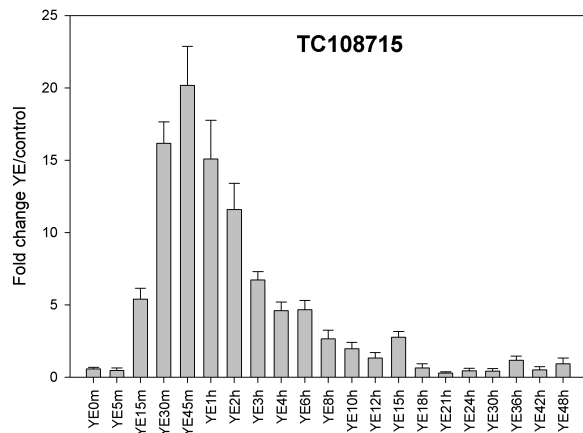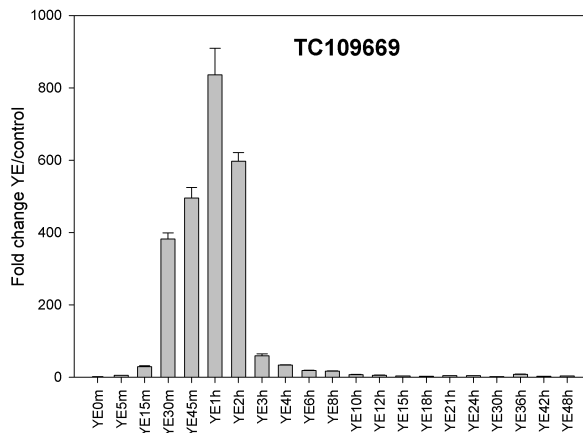

Supplement: Additional file 4 — Semi-quantitative RT-PCR analysis of WRKY transcript levels. The data show representative changes of WRKY transcripts in response to yeast elicitation based on semi-quantitative RT-PCR analysis. Data represent the fold change in transcript level in response to YE as compared to unelicited control. Error bars indicate standard deviation from three biological replicates. [file 1471-2229-8-132-S4.pdf]

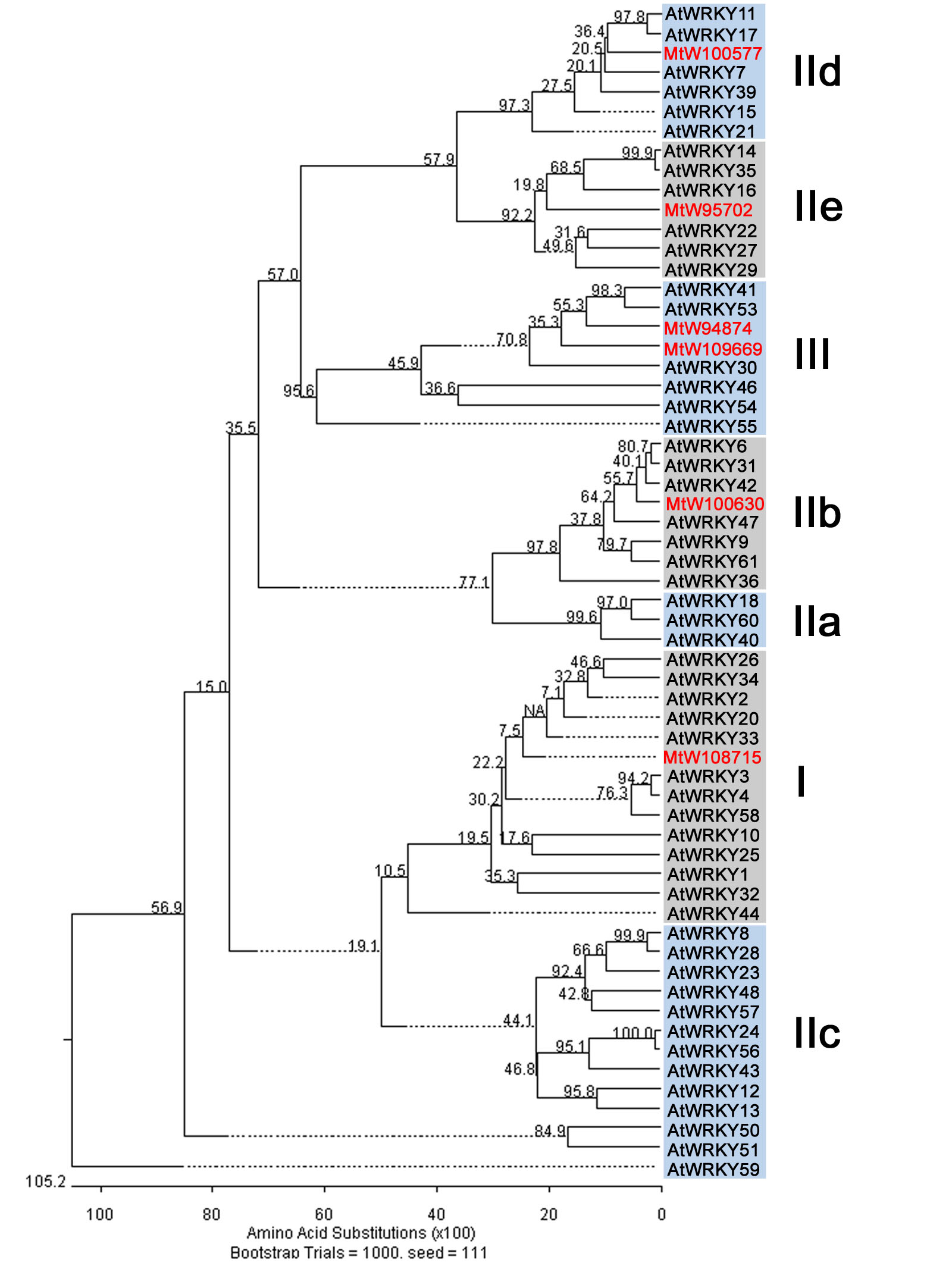

Supplement: Additional file 5 — Phylogenetic analysis of Arabidopsis and M. truncatula WRKY proteins based on their DNA-binding WRKY domain. This figure shows a phylogenetic tree of Arabidopsis and M. truncatula WRKY proteins, based on their DNA-binding WRKY domains. The amino acid sequences of the Medicago WRKY sequences reported here were compared with those of published Arabidopsis WRKY TFs [17] and additional sequences available online [99]. Amino acid sequences from the single WRKY domain of group II and III members or the C-terminal WRKY domain of group I members were aligned using the MegAlign program in the DNASTAR Lasergene package software (DNASTAR, Inc., Madison, WI). The ClustalW method with BLOSUM series of protein weight matrix was used for alignment. The numbers above the branches are bootstrap values from 1000 replicates. [file 1471-2229-8-132-S5.jpeg]

**A**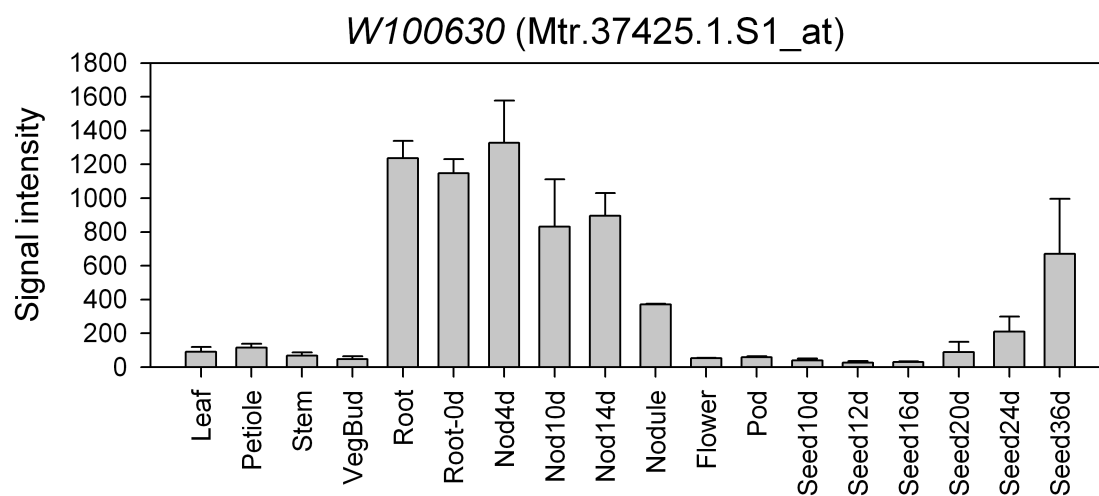**B**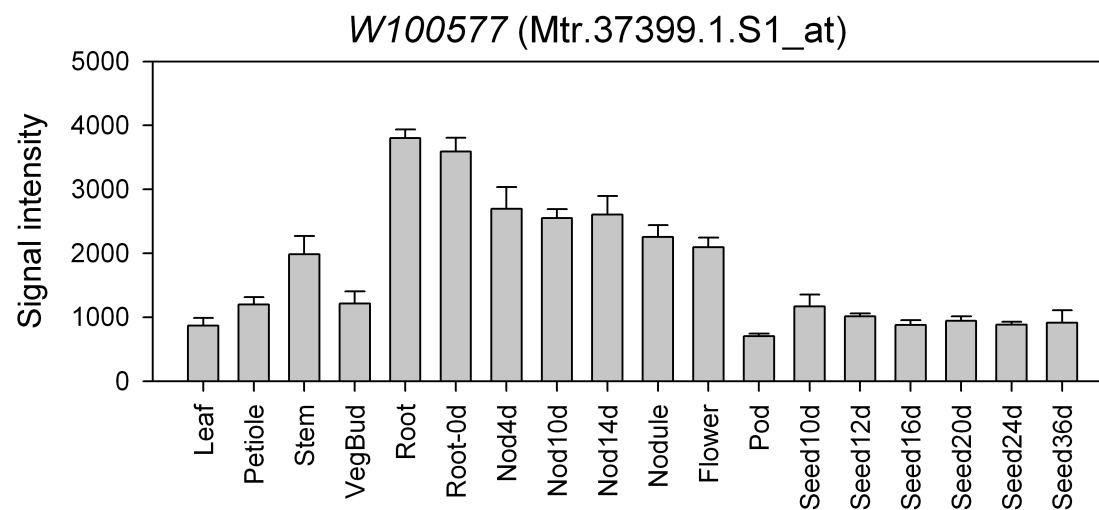**C**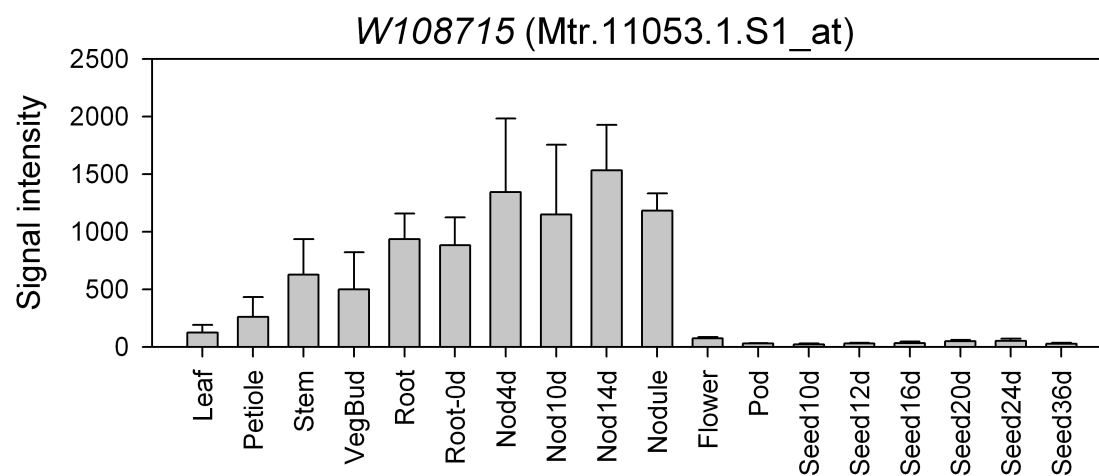**D**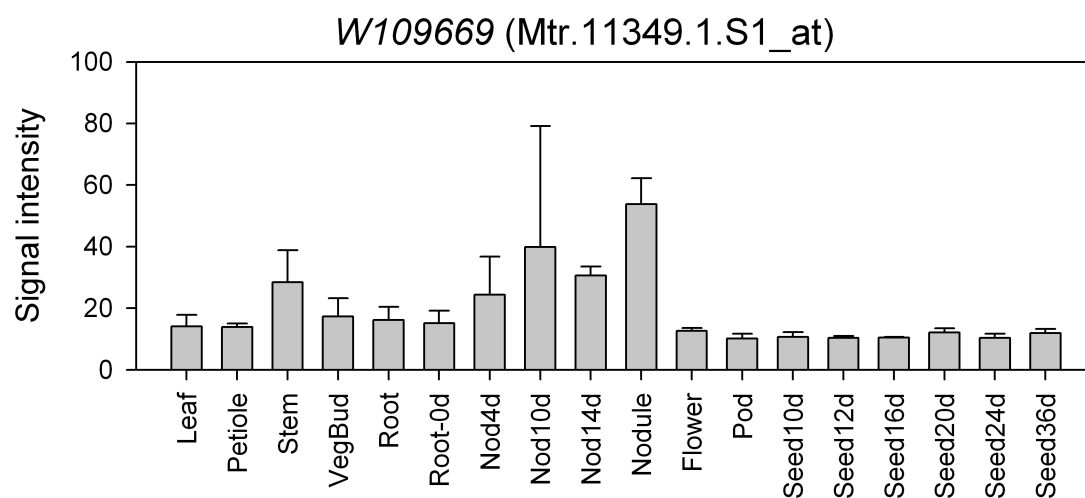

Supplement: Additional file 6 — Affymetrix microarray analysis of the tissue specificity of expression of WRKY TFs. This figure shows WRKY gene expression profiles in different naïve M. truncatula tissues. Genes were: (A) W100630; (B) W100577; (C)W108715; (D)W109669. Transcript levels were measured in the different tissues shown, including seeds at various stages of development (numbers refer to days post pollination, dpp) and nodules (Nod) derived from Rhizobium-inoculated roots at various times (numbers refer to days post-inoculation, dpi). Root-0d – roots at 0 dpi (control for nodule developmental series). Nodule – nodules from 4 weeks old plant. VegBud – vegetative buds (apical and lateral meristem regions). Error bars indicate standard deviation for three biological replicates. Data were mined from the Medicago Gene Atlas [34]. [file 1471-2229-8-132-S6.pdf]

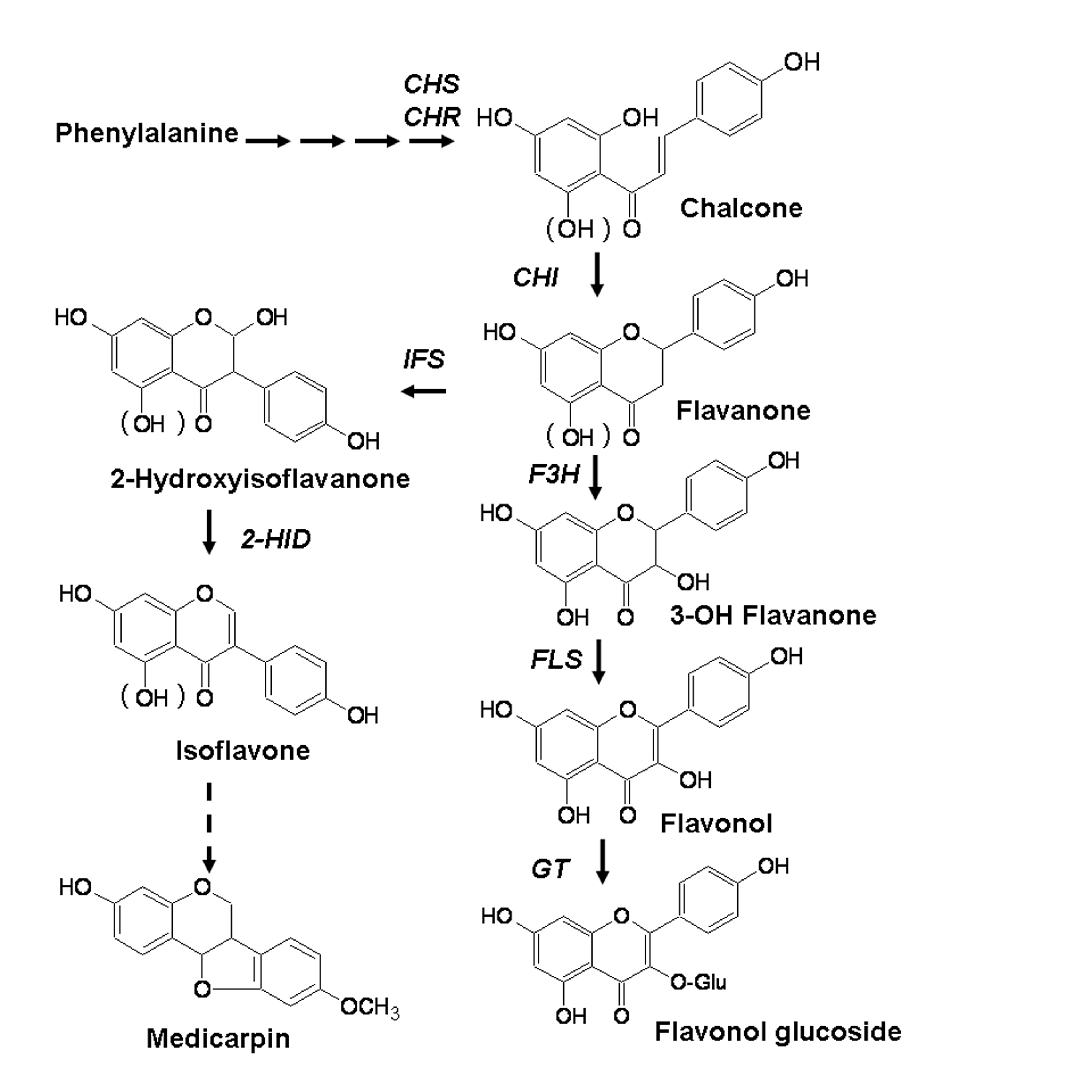

Supplement: Additional file 7 — Scheme of the flavonol biosynthesis pathway. This figure shows a scheme of the flavonol biosynthesis pathway in Medicago. Enzymes are: CHS, chalcone synthase; CHR, chalcone reductase; F3H, flavanone-3-hydroxylase; IFS, isoflavone synthase; 2HID, 2-hydroxyisoflavanone dehydratase; FLS, flavonol synthase; GT, glucosyltransferase. [file 1471-2229-8-132-S7.tiff]

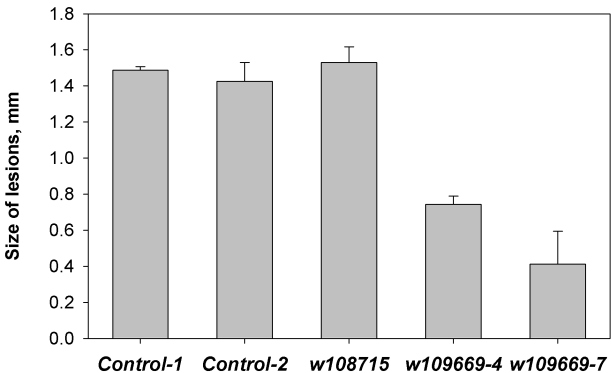

Supplement: Additional file 9 — Enhanced TMV resistance in transgenic tobacco lines overexpressing W109669. The data shown an analysis of the sizes of the secondary lesions formed in transgenic tobacco lines overexpressing W109669 after inoculation with tobacco mosaic virus. Bars show the size (diameter) of secondary lesions on TMV infected control and transgenic tobacco lines expressing Medicago W108715 or W109669. Control plants harbored pBI121. Error bars indicate standard errors for the size of lesions from three control and transgenic lines of the T1 generation. [file 1471-2229-8-132-S9.pdf]
