# Supplementary material for: Elicitor-induced transcription factors for metabolic reprogramming of secondary metabolism in Medicago truncatula
Source: BMC Plant Biol. 2008 Dec 22;8:132. doi: 10.1186/1471-2229-8-132 (PMC2628384; doi:10.1186/1471-2229-8-132)
Supplement: Additional file 8 — Affymetrix analysis of M. truncatula genes involved in the lignin pathway that are induced in response to YE or MJ. This table shows Affymetrix microarray analysis of genes involved in the lignin pathway which were either up-regulated or down-regulated in M. truncatula cell cultures exposed to either yeast elicitor or methyl jasmonate. [file 1471-2229-8-132-S8.doc]

| **Probesets** | **Y2h** | **Y24h** | **M2h** | **M24h** | **Accessions** | **References** |
| --- | --- | --- | --- | --- | --- | --- |
| ***Cinnamic acid 4-hydroxylase (C4H)*** | | |  |  |  |  |
| Mtr.45885.1.S1_at | 2.44 | 1.34 | 1.01 | 0.90 | 1010.m00010 (TC106704) | [1, 2] |
| ***Hydroxycinnamoyl CoA: shikimate hydroxycinnamoyl transferase (HCT)*** | | | | | |  |
| Mtr.40238.1.S1_at | 2.63 | 1.52 | 1.66 | 1.77 | TC106825 | [2, 3] |
| ***Coumarate 3-hydroxylase (C3H)*** | | |  |  |  |  |
| Mtr.43183.1.S1_at | 1.94 | 1.60 | 0.70 | 0.90 | TC94739 | [1, 2] |
| ***Ferulate 5-hydroxylase (F5H)*** | |  |  |  |  |  |
| Mtr.20710.1.S1_at | 0.65 | 1.08 | 1.14 | 2.64 | 1121.m00004 (TC111806) | 77% nucleotide identity to F5H-K10 [1, 2] |
| Mtr.42553.1.S1_s_at | 1.38 | 1.15 | 1.14 | 3.71 | 1121.m00004 (TC111806) | 77% nucleotide identity to F5H-K10 [1, 2] |
| Msa.1740.1.S1_at | 1.51 | 2.74 | 1.95 | 8.05 | iMsa.1740 | 98% nucleotide identity to F5H-K10 [1, 2] |
| Mtr.10628.1.S1_at | 1.37 | 4.75 | 1.62 | 19.60 | TC107424 | 94% nucleotide identity to F5H-K10 [1, 2] |
| ***Caffeic acid 3-O-methyltransferase (COMT)*** | | | |  |  |  |
| Mtr.8599.1.S1_s_at | 2.65 | 1.71 | 1.47 | 1.70 | TC100776 | 81% amino acid homology to COMT [4] |
| Mtr.8599.1.S1_at | 2.63 | 2.01 | 1.46 | 1.63 | TC100776 | 81% amino acid homology to COMT [4] |
| ***Caffeoyl-CoA O-methyltransferase (CCOMT)*** | | | |  |  |  |
| Mtr.16956.1.S1_at | 3.89 | 2.30 | 0.68 | 2.08 | 890.m00022 (TC94321) | 99% amino acid identity to CCOMT [5] |
| ***Cinnamoyl coenzyme A reductase (CCR)*** | | |  |  |  |  |
| Mtr.37520.1.S1_at | 11.47 | 1.11 | 2.39 | 0.98 | TC100838 | 73% amino acid homology to CCR related AT5G14700 [Arabidopsis thaliana] |
| Mtr.5789.1.S1_s_at | 8.47 | 1.35 | 5.31 | 1.57 | BF649727 (AC174319_25) | 88% amino acid homology to CCR [Eucalyptus gunnii] [6] |
| Mtr.37453.1.S1_at | 5.36 | 1.32 | 3.68 | 1.41 | TC100678 (AC174319_25) | 88% amino acid homology to CCR [Eucalyptus gunnii] [6] |
| ***Cinnamyl alcohol dehydrogenase (CAD)*** | | | |  |  |  |
| Mtr.27096.1.S1_at | 4.00 | 2.06 | 1.18 | 1.00 | AW559294 | 69% amino acid homology (partial) to CAD [Eucalyptus gunnii] [7] |
| **Laccases** |  |  |  |  |  |  |
| Mtr.42734.1.S1_at | 3.65 | 1.01 | 1.16 | 0.97 | TC112285 | 89% amino acid homology (partial) to Laccase [Populus trichocarpa] [8] |
| Mtr.9478.1.S1_at | 23.42 | 6.23 | 4.22 | 13.28 | TC103474 | 65% amino acid homology to Lac2 [Pinus taeda] [9] |
| Mtr.12502.1.S1_at | 6.83 | 2.19 | 0.63 | 0.39 | TC95022 | 66% amino acid homology to Lac4 [Pinus taeda] [9] |
| Mtr.1693.1.S1_at | 6.08 | 1.84 | 0.71 | 0.61 | AW736526 | Laccase-like, partial (10%) |
| Mtr.41285.1.S1_at | 2.22 | 0.61 | 1.16 | 0.63 | TC109080 (CT009652_16) | 81% amino acid homology to Lac3 gene ID 817571 [Arabidopsis thaliana] |
| ***Peroxidases*** |  |  |  |  |  |  |
| Mtr.34118.1.S1_at | 62.94 | 0.44 | 0.83 | 22.05 | BQ138499 (AC202373_15) |  |
| Mtr.32367.1.S1_at | 34.89 | 7.73 | 1.97 | 1.21 | AW689446 |  |
| Mtr.46036.1.S1_at | 28.12 | 1.57 | 2.10 | 4.36 | 773.m00015 |  |
| Mtr.42141.1.S1_at | 17.45 | 1.96 | 1.28 | 0.77 | TC110836 |  |
| Mtr.51817.1.S1_s_at | 13.37 | 1.54 | 0.99 | 1.02 | 896.m00007 |  |
| Mtr.42141.1.S1_s_at | 11.96 | 1.87 | 1.25 | 0.74 | TC110836 |  |
| Mtr.10958.1.S1_at | 7.99 | 2.04 | 0.78 | 2.13 | TC108446 |  |
| Mtr.11354.1.S1_at | 7.93 | 0.69 | 0.76 | 0.54 | TC109684 (AC199762_12) |  |
| Mtr.8653.1.S1_at | 6.48 | 3.44 | 1.69 | 2.01 | TC100927 |  |
| Mtr.5966.1.S1_at | 6.42 | 2.32 | 1.15 | 0.96 | BG454205 |  |
| Mtr.38808.1.S1_at | 6.19 | 0.65 | 1.86 | 0.81 | TC103581 |  |
| Mtr.27948.1.S1_at | 4.98 | 0.88 | 1.17 | 0.49 | BF521344 (AC199762_13) |  |
| Mtr.40911.1.S1_s_at | 4.88 | 0.66 | 0.37 | 0.02 | TC108315 (AC187465_18) |  |
| Mtr.2922.1.S1_at | 4.39 | 1.94 | 2.42 | 1.63 | CA922949 |  |
| Mtr.40970.1.S1_at | 4.21 | 1.69 | 1.04 | 2.64 | TC108447 |  |
| Mtr.10372.1.S1_at | 3.80 | 4.14 | 0.78 | 0.49 | TC106546 |  |
| Mtr.15484.1.S1_at | 3.01 | 1.21 | 1.65 | 0.37 | 788.m00010 (TC95986) |  |
| Mtr.5705.1.S1_at | 2.72 | 0.97 | 0.22 | 0.80 | BF645232 |  |
| Mtr.40131.1.S1_at | 2.63 | 2.35 | 4.43 | 3.38 | TC106567 |  |
| Mtr.43507.1.S1_at | 2.52 | 4.26 | 0.72 | 0.69 | TC95489 |  |
| Mtr.40132.1.S1_at | 2.27 | 1.34 | 3.70 | 2.17 | TC106568 |  |
| Mtr.35947.1.S1_at | 2.21 | 3.54 | 2.67 | 0.10 | TC99856 |  |
| Mtr.52185.1.S1_at | 2.00 | 1.32 | 1.44 | 2.77 | 852.m00013 (TC102707) |  |
| Mtr.11242.1.S1_at | 1.97 | 3.04 | 1.69 | 0.23 | TC109333 |  |
| Mtr.34600.1.S1_at | 1.94 | 1.15 | 0.28 | 0.59 | BQ153191 |  |
| Mtr.39716.1.S1_at | 1.74 | 1.12 | 0.30 | 0.48 | TC105551 |  |
| Mtr.8179.1.S1_at | 1.68 | 1.13 | 1.05 | 0.17 | BQ156680 (AC159535_9) |  |
| Mtr.34465.1.S1_s_at | 1.66 | 3.70 | 0.96 | 0.01 | BQ148601 |  |
| Mtr.10370.1.S1_at | 1.60 | 2.05 | 1.01 | 0.59 | TC106540 |  |
| Mtr.40091.1.S1_at | 1.54 | 3.14 | 0.94 | 0.30 | TC106477 |  |
| Mtr.40120.1.S1_at | 1.40 | 1.14 | 0.47 | 0.65 | TC106543 |  |
| Mtr.10356.1.S1_at | 1.38 | 2.39 | 1.02 | 0.41 | TC106484 |  |
| Mtr.40124.1.S1_at | 1.30 | 0.44 | 1.48 | 0.26 | TC106556 |  |
| Mtr.43371.1.S1_at | 1.29 | 1.49 | 1.41 | 2.12 | TC95164 |  |
| Mtr.25211.1.S1_s_at | 1.28 | 0.55 | 0.54 | 2.40 | 1798.m00032 (TC107261) |  |
| Mtr.43159.1.S1_at | 1.15 | 0.76 | 3.29 | 5.65 | TC94676 (AC199762_11) |  |
| Mtr.7078.1.S1_at | 1.07 | 0.48 | 0.92 | 0.61 | CX539193 (AC151725_16) |  |
| Mtr.12914.1.S1_at | 0.94 | 0.99 | 0.65 | 0.16 | TC96382 (AC170990_27) |  |
| Mtr.31347.1.S1_at | 0.85 | 2.10 | 1.60 | 2.80 | AJ504367 (CT573051_36) |  |
| Mtr.7102.1.S1_s_at | 0.79 | 1.08 | 1.12 | 0.14 | CX540478 (AC154090_1) |  |
| Mtr.10969.1.S1_at | 0.78 | 1.23 | 2.59 | 1.07 | TC108472 (CR955005_21) |  |
| Mtr.12276.1.S1_at | 0.77 | 0.98 | 1.07 | 0.16 | TC94210 (AC202500_2) |  |
| Mtr.8654.1.S1_at | 0.76 | 2.12 | 1.24 | 1.96 | TC100928 |  |
| Mtr.9899.1.S1_at | 0.73 | 2.13 | 1.06 | 0.14 | TC104806 |  |
| Mtr.7245.1.S1_at | 0.72 | 1.62 | 0.91 | 0.13 | TC104398 |  |
| Mtr.37542.1.S1_at | 0.65 | 1.52 | 0.86 | 0.10 | TC100885 |  |
| Mtr.44569.1.S1_at | 0.57 | 0.59 | 0.71 | 0.06 | TC97623 |  |
| Mtr.51089.1.S1_at | 0.44 | 0.95 | 7.89 | 5.08 | 721.m00010 (TC102451) |  |
| Mtr.15379.1.S1_at | 0.32 | 2.60 | 0.46 | 0.12 | 784.m00005 (TC101565) |  |

1. Reddy MS, Chen F, Shadle G, Jackson L, Aljoe H, Dixon RA: **Targeted down-regulation of cytochrome P450 enzymes for forage quality improvement in alfalfa (Medicago sativa L.)**. *Proc Natl Acad Sci U S A* 2005, **102**(46):16573-16578.

2. Chen F, Srinivasa Reddy MS, Temple S, Jackson L, Shadle G, Dixon RA: **Multi-site genetic modulation of monolignol biosynthesis suggests new routes for formation of syringyl lignin and wall-bound ferulic acid in alfalfa (Medicago sativa L.)**. *Plant J* 2006, **48**(1):113-124.

3. Shadle G, Chen F, Srinivasa Reddy MS, Jackson L, Nakashima J, Dixon RA: **Down-regulation of hydroxycinnamoyl CoA: shikimate hydroxycinnamoyl transferase in transgenic alfalfa affects lignification, development and forage quality**. *Phytochemistry* 2007, **68**(11):1521-1529.

4. Gowri G, Bugos RC, Campbell WH, Maxwell CA, Dixon RA: **Stress Responses in Alfalfa (Medicago sativa L.): X. Molecular Cloning and Expression of S-Adenosyl-l-Methionine:Caffeic Acid 3-O-Methyltransferase, a Key Enzyme of Lignin Biosynthesis**. *Plant Physiol* 1991, **97**(1):7-14.

5. Inoue K, Sewalt VJ, Murray GB, Ni W, Sturzer C, Dixon RA: **Developmental expression and substrate specificities of alfalfa caffeic acid 3-O-methyltransferase and caffeoyl coenzyme A 3-O-methyltransferase in relation to lignification**. *Plant Physiol* 1998, **117**(3):761-770.

6. Lacombe E, Hawkins S, Van Doorsselaere J, Piquemal J, Goffner D, Poeydomenge O, Boudet AM, Grima-Pettenati J: **Cinnamoyl CoA reductase, the first committed enzyme of the lignin branch biosynthetic pathway: cloning, expression and phylogenetic relationships**. *Plant J* 1997, **11**(3):429-441.

7. Goffner D, Van Doorsselaere J, Yahiaoui N, Samaj J, Grima-Pettenati J, Boudet AM: **A novel aromatic alcohol dehydrogenase in higher plants: molecular cloning and expression**. *Plant Mol Biol* 1998, **36**(5):755-765.

8. Ranocha P, McDougall G, Hawkins S, Sterjiades R, Borderies G, Stewart D, Cabanes-Macheteau M, Boudet AM, Goffner D: **Biochemical characterization, molecular cloning and expression of laccases - a divergent gene family - in poplar**. *Eur J Biochem* 1999, **259**(1-2):485-495.

9. Sato Y, Wuli B, Sederoff R, Whetten R: **Molecular Cloning and Expression of Eight Laccase cDNAs in Loblolly Pine (Pinus taeda).** *J Plant Res* 2001, **114**(1114):147-155.
